# Experiments upon Digestion

**Published:** 1857-07

**Authors:** Francis G. Smith

**Affiliations:** Professor of Institutes of Medicine in the Medical Department of Pennsylvania College


ART. II.—*Experiments upon Digestion.* By FRANCIS G. SMITH, M.D., Professor of Institutes of Medicine in the Medical Department of Pennsylvania College.

IN the Medical Examiner for July and September, 1856, the writer published some experiments performed upon Alexis St. Martin, with the view of determining the nature of the acid of the gastric juice, and the influence of the latter fluid upon various alimentary substances. The conclusions arrived at were, that the acid reaction is due to the presence of *lactic acid;* that nitrogenized food is converted into albuminose; that fatty articles undergo no change beyond minute division; that *amylaceous,* or *starchy materials* (in man) *are converted into glucose* or *grape sugar.*

To the last of these conclusions the objection has been urged, that the glucose manifested in the products of digestion *"existed beforehand in the bread used as food."**

* Prof. J. C. Dalton, in Amer. Jour. of Med. Sci., October, 1856.

To those who did not witness the experiments, such an objection may appear well grounded, particularly as it is well known that glucose is formed in the process of panification; but with those who were present, and who saw the evidences of a much larger quantity of glucose in the fluid of digestion than they ever met with in the examination of bread alone, such objections have less weight.

Granting, however, the justice of the criticism, and feeling great respect for the source from whence it came, but at the same time contending for truth alone, the writer has, during the past month, performed a corroborative experiment, with the assistance of his distinguished friend, Dr. E. Brown-Sequard, who kindly *lent himself* for the purpose, in place of St. Martin, unfortunately no longer within reach.

Dr. Brown-Sequard has, as Montegre had, the faculty of vomiting at will, and thus exhibiting the condition of alimentary articles at any stage of digestion. With the view of determining the action of gastric juice upon amylaceous food, he lent his valuable aid and experimental tact in the following researches.

After a night's fasting, the stomach was thoroughly cleansed by copious draughts of water, and a portion of plain arrowroot, carefully washed before boiling, and subsequently tested for glucose (of which not a trace was visible), was administered. After about thirty minutes had elapsed, the contents of the stomach were ejected by a simple spasmodic contraction, and without nausea. The fluid was distinctly acid to litmus, and gave a copious precipitate of red suboxide of copper, with Trommer's test.

The process was continued with subsequent ejections for nearly an hour, with the same result on each repetition, with the exception that the quantity of glucose detected was, if anything, increased. The fluid was not tested for starch or dextrine.

A portion of the same arrowroot was treated with saliva, and allowed to stand about the same length of time that the previous portions had remained in the stomach. Trommer's test revealed glucose, but by no means in so large a quantity as in the previous experiments.

It may be urged by some that these results are not reliable, inasmuch as the process of digestion was not healthily performed. To this it is answered, that there was no evidence of disorder in the function other than the spasmodic contraction alluded to, and that distinguished physiologists have placed implicit confidence in the experiments of Montegre* and others, of a similar character. Taken in connection with the experiments

* Adelon and Chaussier, art. "Digestion," in Dict. des Sciences Méd., t. ix, pp. 422–3; Stevens, De Alimentorum Concoctione; Spallanzani, Exper. sur la Digestion.

upon St. Martin, therefore, this one seems to bear out the conclusion then arrived at, that "starchy materials are digested in the human stomach; that human gastric juice does not prevent the conversion of starch into grape sugar; and that this conversion may take place in the stomach independently of the action of saliva." In regard to the last point, it may be observed that the fluid character of the food obviated the necessity for insalivation, as Bernard's experiments have shown, and that the glycogenic change, therefore, could have been but little, if at all, influenced by saliva, the effects of which are declared by Bernard to be nullified by the acid reaction of the gastric juice.

